# Supplementary material for: Interleukin-13 rs1800925/-1112C/T promoter single nucleotide polymorphism variant linked to anti-schistosomiasis in adult males in Murehwa District, Zimbabwe
Source: PLoS One. 2021 May 28;16(5):e0252220. doi: 10.1371/journal.pone.0252220 (PMC8162643; doi:10.1371/journal.pone.0252220)
Supplement: S1 Table — (DOCX) [file pone.0252220.s001.docx]

| **IL-13 – 1055 C/T allele/genotype** | **Frequencies in population** | | **Hardy-Weinberg Equilibrium**  **Test *X* ^2^ (p value)** |
| --- | --- | --- | --- |
|  | Expected | Observed |  |
| **C** | 79.5 | 66 | 5.429 (0. 020) |
| **T** | 57.5 | 71 |  |
| **CC** | 66 | 121 | 40.736 (0.000) |
| **CT** | 219 | 168 |  |
| **TT** | 71 | 66.9 |  |
