# Supplementary material for: Interleukin-13 rs1800925/-1112C/T promoter single nucleotide polymorphism variant linked to anti-schistosomiasis in adult males in Murehwa District, Zimbabwe
Source: PLoS One. 2021 May 28;16(5):e0252220. doi: 10.1371/journal.pone.0252220 (PMC8162643; doi:10.1371/journal.pone.0252220)
Supplement: S2 Table — (DOCX) [file pone.0252220.s002.docx]

| **Villgaes** | **Dombwe**  n (%) | **Jekwa**  n (%) | **Mutize**  n (%) | **Kapasura**  n (%) | **Kareza**  n (%) | **Magaya**  n (%) | **Guzha**  n (%) | **Inyagui**  n (%) | | **Total** | |
| --- | --- | --- | --- | --- | --- | --- | --- | --- | --- | --- | --- |
| **< 4 ng/mL** | 10 (5.1) | 25 (12.8) | 12 (6.2) | 34 (17.4) | 22 (11.3) | 44 (22.6) | 31 (15.9) | 17 (8.7) | 195 (100) | |  |
| **> 4 ng/mL** | 1 (12.5) | 0 (0.0) | 2 (25.0) | 1 (12.5) | 1 (12.5) | 1 (12.5) | 0 (0.0) | 2 (25.0) | 8 (100) | |  |
