# Supplementary material for: Interleukin-13 rs1800925/-1112C/T promoter single nucleotide polymorphism variant linked to anti-schistosomiasis in adult males in Murehwa District, Zimbabwe
Source: PLoS One. 2021 May 28;16(5):e0252220. doi: 10.1371/journal.pone.0252220 (PMC8162643; doi:10.1371/journal.pone.0252220)
Supplement: Attachment — Submitted filename: Response to Reviewers.docx [file pone.0252220.s004.docx]

Point-by-point response to the comments raised dated 2 February 2021.

| **Reviewers Comments** | **Response** | **Section; Page, line number** |
| --- | --- | --- |
| **Reviewer #1 Comments:**  ***The study attempted to evaluate the IL-13 in susceptibility or resistance against schistosomiasis and association to risk of prostate cancer development. The study should provide promising information on understanding the role of the cytokine in host response to the disease schistosomiasis.***  ***Introduction***  *Line 99-100 "Host genetic variability of encoding genes such as polymorphisms located in the promoter region modify gene transcription and cytokine production in parasitic and autoimmune diseases" is not explicit and appeared inconclusive, please rephrase.*  *Line 108-110 add reference to the statement "Single nucleotide polymorphisms (SNPs) are the most common genetic variations..... sequences and protein structure (ref.)*  *Line 116- Isnard et al (2011)... and not Isnard et al., in 2011*  *Line 144- Remove schistosome from the statement "S. haematobium schistosome egg burden was associated"*  ***Materials and Methods***  *Line 164- Inclusion criteria: Reason on focusing on adults aged 18 and above should be clearly stated.*  *Line 174-175: Schistosome infected individuals were treated with praziquantel(PZQ) at the standard single oral dose of 40mg/kg per body weight (ref.)*  *Line 162-163: Detailed description of study areas was not well provided in this study. Providing map, daily activities of the study population will help*  *Line 360-362: "Binomial logistic regression was used to predict the risk of prostate cancer development between participants with prostate specific antigen levels > 4 ng/mL and < 4ng/mL using IL362 13 cytokine concentrations and schistosomiasis status as the predictor value". The statement is more of methodology than results, rephrase.*  ***Discussion:***  ***The discussion is well written but more recent literatures on study should be included to help support the outcome of the present study.*** | - We are appreciative for the acknowledgement on the topic we are reporting on. - We have revised the sentence to read as follows: *Host genetic variability such as single nucleotide polymorphisms located in the promoter region of encoding genes* ***may*** *modify gene transcription and cytokine production in parasitic infections and autoimmune diseases.* - We have included the reference: 31. *Howard TD, Whittaker PA, Zaiman AL, Koppelman GH, Xu J, Hanley MT et al. Identification and association of polymorphisms in the interleukin-13 gene with asthma and atopy in a Dutch population. Am J Respir Cell Mol Biol. 2001;25(3):377–84. PMID: 11588017 doi: 10.1165/ajrcmb.25.3.4483.* - The reference has been fixed. - Schistosome has been removed from the statement. - The reason on the for focusing on adults aged 18 years and above is because the study is a sub-study of prostate cancer and schistosomiasis cross-sectional study that included only male adult participants aged 18 years and above. Adults aged 18 years and above were included into the study because they were able to understand the study and voluntarily give informed consent. - Reference has been provided: 58. Crompton DWT, World Health Organization. Preventative chemotherapy in human helminthiasis: coordinated use of anthelminthic drugs in control interventions: a manual for health professionals and programme managers. World Health Organization 2006. Available at: <https://apps.who.int/iris/handle/10665/43545>. - Even though we have not provided a map we have added information on the study population and area: *The study area was chosen because it has a high schistosomiasis burden of 47.4 % in school aged children a representative of the study population [52]. Overall population of Murehwa District is 199 607 and about 94 000 individuals are men [53]. The District consists of more than 90 % of the area is rural where majority of the residents rely on nearby rivers for their domestic needs and farming activities. Sanitation in the area is poor with the majority of residents relying on unsafe drinking water and open defecation. The main study assessed possibility of prostate cancer development due to schistosome infections hence, only male adult individuals were enrolled.* - Agreed and statement has been rephrased to read as follows: ‘*Risk of prostate cancer development between participants with prostate specific antigen levels > 4 ng/mL and < 4ng/mL assessed by IL-13 cytokine concentrations and schistosomiasis status was insignificant.’* - We appreciate the acknowledgement and we included the limited available information in the discussion on the following information: account for low number of schistosomiasis infected participants, low infection intensities, information on other inflammatory mediators between prostatic inflammation and prostate carcinogenesis and the study limitations. | **Page 4; Lines 96-98**  **Page 5; Lines 121-122**  **Page 5; Lines 123.**  **Page 6; Line 136**  **Pages 7; lines 171-172 and page 8; lines 185-186**  **Page 9; Line 218**  **Page 7 and 8; Lines 174-181**  **Page 16; Lines 390-392**  **Page 17, page 20 and page 21** |
| **Reviewer # 2 Comments:**  ***The manuscript is technically sound and the data support the conclusion. However, the study looks like two different studies (one on the association between IL-13rs1800925/-1112C/T promoter SNPs and Schistosoma haematobium infection, and the other on association between IL-13rs1800925 polymorphism and prostate-specific antigen levels) but the nexus between the two does not come out clearly in the literature review and discussion.***  ***The author should also consider and mention that adult humans do not shed S. haematobium eggs well in urine and urinary egg counts may therefore not be a good indicator of infection intensity or disease burden in adults. In addition it is noteworthy that although IL-13 is an important player in schistosomiasis carcinogenesis, there are other important players such as infiltrin which should have been mentioned in discussion.***  ***Being a cross-sectional study, no influence could have been detected and therefore the word "influence" in lines 34 and 152 should be changed to "association".***  ***The analyses were performed appropriately and rigorously however, the power of the study could have been compromised by the small number of infected individuals (n=50). The data underlying the findings were made available but it would have been better if the age distribution of the subjects was also presented.***  ***The manuscript is presented in an intelligible fashion and in standard English. However, there are several grammatical errors and omissions that should be corrected. For example, the term "schistosomiasis infected" should be changed to "S. haematobium-infected"*.** | - Thank you and we agree on the assessment. We have now included additional information to demonstrate the nexus between the IL-13 SNPs or concentration, schistosomiasis and prostate specific antigen. Please note that the association of IL-13 SNPs, concentrations, schistosomiasis and prostate specific antigen has very limited literature hence, we believe that our manuscript will build literature on the subject.   *‘Screening for prostate cancer is initially done by using prostate-specific antigen levels to detect the diseases early stage for better management and reduction of disease specific mortality. Whilst prostate specific-antigen levels above 4.0 ng/mL serves as a reference point for further prostate management. To further determine risk of prostate cancer, combination of prostate specific antigen with single nucleotide polymorphisms was shown to be effective in men with prostate specific antigen levels greater than 4 ng/mL. Combination of genetics and the PSA test is useful for predicting the risk of prostate cancer that enables stratifying the population into different risk groups that may be a basis for the development of personalized screening for prostate cancer.*   - Thank you very much, we appreciate the input and have included the information in the manuscript such as the macrophage inhibitory cytokine-1, IL-6, tumour necrosis alpha, *S. haematobium*, egg secreted infiltrin protein, IL-10 and IL-8      - The word influence has been changed to association. - We agree with the reviewer and we have now included a table (Table 1) with the age distribution of the study participants. - All authors have proofread the manuscript to correct grammatical errors. | **Page 6; Lines 140-148**  **Page 18; lines 408- 410, Page 20; Lines 463-473 Page 21; lines 476-478**  **Lines 153 and 39**  **Page 14; Line 331** |
| **Reviewer # 3 Comments:**  ***ABSTRACT*** *Page 2: Lines 32-34 The authors should correct "Our study evaluated the frequency of the IL-13 rs1800925/-1112 C/ T promoter single nucleotide polymorphisms (SNPs) and assessed the influence of the variants on IL-13 cytokine levels." to read "Our study evaluated the frequency of the IL-13 rs1800925/-1112 C/ T promoter single nucleotide polymorphisms (SNPs) among schistosomiasis infected individuals and assessed the influence of the variants on IL-13 cytokine levels."*  *Page 2: Lines 46-48 The authors should indicated whether the sentence "There were significantly (p<0.05) higher IL-13 cytokine levels among participants with the genotypes CC and CT; median 92.25 pg/mL and 106.5 pg/mL, respectively, compared to TT variant individuals; 44.78 pg/mL." is with respect to schistosomiasis infected individuals or uninfected.*  ***INTRODUCTION***  *Page 4: Line 82 Please restructure "...Schistosomiasis is a neglected parasitic tropical disease" to read "...Schistosomiasis is a neglected tropical parasitic disease"  Page 4: Line 91 Please restructure "...Schistosomiasis acute infections" to read "...Acute schistosomiasis infections"*  *Page 4: Lines 98-101 Needs grammatical restructuring.*  *Page 5: Line 109 Please restructure "...located in the coding regions of the genes" to read "...located in the coding regions of genes"*  *Page 5: Lines 122-125 The sentence "In contrast, to the above findings recently Adedokun et al. in 2018 found no statistical difference in the IL-13 rs7719175 genotypic or allelic frequencies between schistosome-infected and uninfected controls or any association with disease [26]." would be better in the Discussion Section.*  *Page 5: Lines 125-127 The sentence "Furthermore, no association between IL-13 rs1800925 C/T and autoimmune diseases such as Graves’ disease risk [27] and rheumatoid arthritis [18] has been established." should be removed because it does not appear to have any relevance there.*  *Page 6: Lines 150 and 151 The sentence "Therefore, the aim of the study were..." should be corrected to read "Therefore, the aim of the study was...".*  ***MATERIALS AND METHODS***  *Page 7: Lines 161 The authors should also give a description of the enrolment or case definition of participants with prostate cancer.*  ***RESULTS***  *Page 12: Lines 281-282 It was observed that certain data presented in this study had earlier been published by the authors [Choto et al. Infectious Agents and Cancer (2020) 15:59; PMID: 33042215]. The authors should have made mention of this in this section.*  *Page 12: Lines 285-285 The authors had earlier indicated that 366 participants were enrolled but genotypic results for IL-13 -1112C/T polymorphism were given for only 356 participants. The authors should give reasons for this discrepancy.*  *Page 13: Line 308 The authors presented IL-13 cytokine concentrations results for only 107 participants out of the 366 participants that were enrolled. The authors should also give reasons for this discrepancy.*  *Page 15: Line 367 The authors should review this Table to ensure that the Title is a true reflection of the contents.* | - Statement has been corrected to read :‘*Our study evaluated the frequency of the IL-13 rs1800925/-1112 C/ T promoter single nucleotide polymorphisms (SNPs) among schistosomiasis infected individuals and assessed the association of the variants on IL-13 cytokine levels’*. - Statement has been specified to read as follows: *There were significantly (p<0.05) higher IL-13 cytokine levels* ***among both infected and uninfected*** *participants with the genotypes CC and CT; median 92.25 pg/mL and 106.5 pg/mL, respectively, compared to TT variant individuals; 44.78 pg/mL*. - Statement has been corrected. - Statement has been restructured - Statement has been restricted to read as follows: *Schistosome infection associated cytokines are connected to cytokine gene polymorphisms of individual variability that influence immune responses and disease outcomes [11]. Cytokine gene polymorphisms such as single nucleotide polymorphisms located in the promoter region of encoding genes may modify gene transcription and cytokine production in parasitic infections and autoimmune diseases.* - Statement has been restructured accordingly. - We believe the sentence adds value into the introduction by showing differences in the results conducted by other studies hence also validating the need to conduct our study. However, we have included the results of the study to compare to our findings in the discussion section. - We agree with the reviewer and the statement has been removed. - Sentence has been corrected ‘were’ had been replaced with ‘was’ - We have included information on prostate cancer. ‘*Screening for prostate cancer is initially done by using prostate-specific antigen levels to detect the diseases early stage for better management and reduction of disease specific mortality [48]. Whilst prostate specific-antigen levels above 4.0 ng/mL serves as a reference point for further prostate management [49]. To further determine risk of prostate cancer, combination of prostate specific antigen with single nucleotide’.* - Statements have been included in the manuscript to acknowledge the previously presented data by Choto et al. (2020). - Noted and we have included a reason for the discrepancy in the results and participants enrolled in the manuscript. ‘*There was unsuccessful genotyping of 10 samples due to unsuccessful genotyping.*      - Two hundred and fifty-nine (259) samples had **undetectable** IL-13 concentrations hence the IL-13 results are for only 107 samples. - The table title has been revised and it now reads *‘****Table 3: Risk of prostate cancer development (PSA > 4 ng/mL and < 4ng/mL) association to schistosomiasis status, IL-13 cytokine concentration and IL-13 rs1800925 variants*’** | **Page 2; Lines 36-39**  **Page 2 and 3; lines 53-55**  **Page 4; Line 80**  **Page 4; Line 88**  **Page 4 Lines 94-99**  **Page 5; Line 120**  **Page 6; Lines 130-134.**  **Page 18; lines 411-414**  **Page 6; Line 151**  **Page 6; Lines 141-144.**  **Page 7; lines 173 Page 13; line 315.**  **Page 13; Lines 315-316.**  **Page 15; Line 343.**  **Page 15; line 341**  **Page 17; Lines 397.** |
| - **Academic Editor Comments and additional requirements** | | |
| 1. *Please ensure that your manuscript meets PLOS ONE's style requirements, including those for file naming. The PLOS ONE style templates can be found at:*   [*https://journals.plos.org/plosone/s/file?id=wjVg/PLOSOne_formatting_sample_main_body.pdf*](https://journals.plos.org/plosone/s/file?id=wjVg/PLOSOne_formatting_sample_main_body.pdf) *and*  [*https://journals.plos.org/plosone/s/file?id=ba62/PLOSOne_formatting_sample_title_authors_affiliations.pdf*](https://journals.plos.org/plosone/s/file?id=ba62/PLOSOne_formatting_sample_title_authors_affiliations.pdf)    *2. Please provide additional details regarding participant consent. In the ethics statement in the Methods and online submission information, please ensure that you have specified whether consent was informed.*    *3. In the Methods, please clarify: - Why written consent could not be obtained - Whether the Institutional Review Board (IRB) approved use of verbal consent - How verbal consent was documented For more information, please see our guidelines for human subjects research:* [*https://journals.plos.org/plosone/s/submission-guidelines#loc-human-subjects-research*](https://journals.plos.org/plosone/s/submission-guidelines#loc-human-subjects-research)    *4. In your Methods section, please provide additional information about*  *i)the participant recruitment method and*    *ii)the demographic details of your participants.*  *Please ensure you have provided sufficient details to replicate the analyses such as:*  *a) the recruitment date range (month and year),*  *b) a table of relevant demographic details,*  *c)a statement as to whether your sample can be considered representative of a larger population,*  *d) a description of how participants were recruited, and*  *e) descriptions of where participants were recruited and where the research took place.*    *5. Please provide a sample size and power calculation in the Methods,or discuss the reasons for not performing one before study initiation.*    *6. Please ensure you have discussed any potential limitations of your study in the Discussion, including study design, sample size and/or potential confounders.*    *7.We note that you have indicated that data from this study are available upon request. PLOS only allows data to be available upon request if there are legal or ethical restrictions on sharing data publicly. For more information on unacceptable data access restrictions, please see* [*http://journals.plos.org/plosone/s/data-availability#loc-unacceptable-data-access-restrictions*](http://journals.plos.org/plosone/s/data-availability#loc-unacceptable-data-access-restrictions)*.* | - We have tried our best to make sure that the manuscript is compliant with the PLOS ONE guidelines. - We have included the following statement in the ethics section: *Prior to enrolment, the study aims and procedures were explained to all participants in the local language (Shona). Written informed consent was obtained from participants.* - Written consent was obtained. - Participant recruitment information has been provided the methodology section. *Participants from rural villages in Murehwa Disctrict were invited to be part of the study through invitation by the village health workers to report to 8 sampling centres that are used by the community for meetings or health education and immunisation programs (such centres include schools or primary health centres) for enrolment into the study. The sampling centres were Jekwa rural clinic, Dombwe rural clinic, Mutize primary school, Kareza gathering point, Kapasura gathering point, Magaya primary school, Guzha primary school and Inyagui primary school. Participants enrolled into the study provided their age or date of birth, samples for parasitological diagnosis and blood sample for serological assays.*      - Demographic details of the participants have been provided in the results section (Table 1). - Recruitment month and year were included: *November 2019* - Table of demographics details has been included. - *The sample can be considered representative of a larger population in the study area*. The statement is included in the methodology section - We have now provided the information in the methodology section and in the above response to query # 4. - We have now provided the information in the methodology section and in the above response to query # 4. - Sample size calculations have been provided in the methodology section. ‘*A sample size of 245 including 20 % added on to account for drop out was calculated [54] based on the inferred schistosomiasis prevalence for adults of 15.8 % (a third of the 47.4 % for the school aged children)’* - Limitations have been included in the discussion.   *‘There are some limitations observed in this study caused by the low number of schistosomiasis infected participants and single nucleotide polymorphisms locus may not have provided us with a clear understanding of the genetic effects of IL-13 on its cytokine productions and risk of prostate cancer. As a result, more work is recommended for the IL-13 promoter single nucleotide polymorphisms assessments. Lack of confirmed prostate cancer cases could have limited assessment of the association of IL-13 cytokine levels or IL-13 -1112C/T variants and risk of prostate cancer. Further, only few numbers of individuals were found with prostate specific antigen above 4 ng/mL that limited our interpretation of the results’*   - Data has been provided as supporting files (S1 File) | **Page 7; Lines 165-167.**  **Page 7; Lines 165- 167.**  **Page 8; Lines 191- 202.**  **Page 14; Line 332.**  **Page 7; Line 171**  **Page 14; Line 332**  **Page 8; Lines 194**  **Page 8; Lines 191- 202**  **Page 8; Lines 191- 202**  **Page 8; Lines 189-191**  **Page 21; Lines 495-503** |
| - *Specifically, questions about the methodology and study design should be addressed; explanations should be given for why numbers don't tally in your manuscript.* - *You enrolled  366 participants but report SNP results for 356 participants (Page 12: Lines 284-285) and no reason(s) was/were given for the remaining one that was missing; probably due to* *unsuccessful genotyping.* - *Of the 366 participants that were enrolled, IL-13 cytokine concentrations were reported for only 107 participants and no reason(s) was/were given for the remaining unreported results.* - *In addition, the authors did not state how many participants out of the 107 had schistosomiasis and how many were uninfected.* - *It was observed that certain data presented in this study had earlier been published by the authors [Choto et al. Infectious Agents and Cancer (2020) 15:59; PMID: 33042215], but no mention was made about this in the Results section (Page 12: Lines 281-282) so as to avoid any semblance of duplicate publishing.* - *You need to also clarify the connection between the stated aims of your study; not much attention was given to the prostate antigen aspect. The study looks like two different studies (one on the association between IL-13rs1800925/-1112C/T promoter SNPs and Schistosoma haematobium infection, and the other on association between IL-13rs1800925 polymorphism and prostate-specific antigen levels) but the nexus between the two does not come out clearly in the literature review and discussion.* - *There are also grammatical errors that need to be edited.* | - We have addressed the issue in the above response to comments raised by reviewer # 3 - We have addressed the issue in the above responses to comments raised by reviewer # 3 - We have now provided the information in the manuscript. *Participants with schistosomiasis (n = 20) had lower but not significant levels of IL-13, 75.64 (14.52 - 287.50) pg/mL compared to schistosomiasis uninfected group (n = 87) 89.88 (40.03 - 206.20) pg/mL; p = 0.481.* - We have acknowledged the results that were earlier published by Choto et. al (2020). Also, we have included reference in the results section. - We have addressed the query by providing information on the nexus between polymorphisms and prostate specific antigen levels in the above response to comments raised by reviewer # 2. - We have tried our best to revise the manuscript for grammatical errors. | **Page 13; Line 315-316**  **Page 15; Line 343**  **Page 15; Line 345**  **Page 8; line 173**  **Page 13; Lines 315**  **Page 6; Lines 140-148** |
